# Drilling Damage in Composite Material

**DOI:** 10.3390/ma7053802

**Published:** 2014-05-14

**Authors:** Luís Miguel P. Durão, João Manuel R.S. Tavares, Victor Hugo C. de Albuquerque, Jorge Filipe S. Marques, Oscar N.G. Andrade

**Affiliations:** 1 ISEP/CIDEM, School of Engineering-Polytechnic of Porto, Rua Doutor António Bernardino de Almeida, 431, Porto 4200-072, Portugal; E-Mails: 1110974@isep.ipp.pt (J.F.S.M.); oscarnicolau@gmail.com (O.N.G.A.); 2 Instituto de Engenharia Mecânica e Gestão Industrial (INEGI), Departamento de Engenharia Mecânica (DEMec), Faculdade de Engenharia, Universidade do Porto (FEUP), Rua Doutor Roberto Frias, s/n, 4200-465 Porto, Portugal; E-Mail: tavares@fe.up.pt; 3 Programa de Pós-Graduação em Informática Aplicada (PPGIA), Universidade de Fortaleza (UNIFOR), Av. Washington Soares, 1321, Sala J30, CEP 60.811-905, Edson Queiroz, Fortaleza, Brazil; E-Mail: victor.albuquerque@unifor.br

**Keywords:** fiber reinforced composites, carbon fibers, drilling, thrust force, delamination, radiography, computational image processing and analysis, bearing test

## Abstract

The characteristics of carbon fibre reinforced laminates have widened their use from aerospace to domestic appliances, and new possibilities for their usage emerge almost daily. In many of the possible applications, the laminates need to be drilled for assembly purposes. It is known that a drilling process that reduces the drill thrust force can decrease the risk of delamination. In this work, damage assessment methods based on data extracted from radiographic images are compared and correlated with mechanical test results—bearing test and delamination onset test—and analytical models. The results demonstrate the importance of an adequate selection of drilling tools and machining parameters to extend the life cycle of these laminates as a consequence of enhanced reliability.

## 1. Introduction

Reinforced composite laminates are one of the most remarkable families of materials of this technological era. Their ability to be tailored for use and endless possibilities provided by the combination of reinforcements together with their alignment and fiber fraction, allow design engineers to have almost total freedom in the design of new parts. Unique properties such as low weight, high strength and stiffness are normally referred to whenever the advantages of these materials are listed. Nevertheless, some problematical issues remain concerning the use of composite laminates, thus providing arguments for the selection of conventional materials instead of composites, mainly in structural parts. Some of these issues are cost-related, but considerations about reliability or fatigue resistance also cause some difficulties for a wider usage of these materials.

However, the importance of composite materials has been growing steadily over the last decade, which can be confirmed by their intensive use in the new Airbus A380 or Boeing 787 airplanes. In the latter, 50% of the weight of its primary structure will be made of composite materials [1], an unprecedented ratio; which was difficult to imagine just some 30 years ago. One can now find composite materials not only in the aeronautical field, but also in other industries such as automotive, railway, marine or sports goods. There is no doubt that the level of confidence and reliability already achieved in metallic materials can also be reached for composites, it is just a question of time.

Taking the main problematic issues related to laminate parts into account, it is possible to find different arguments for the selection of conventional materials. One of them is associated with the relative complexity and cost of the production process. In the later stage of parts production, machining operations like drilling are frequently needed in composite structures, as the use of bolts, rivets or screws is required to join the parts. Generally, machined parts have poor surface appearance and tool wear is higher. One of the problems related with composites’ machining is the nature of the fibre reinforcement, which is usually very abrasive and causes rapid tool wear and deterioration of the machined surfaces [2]. As early as 1983, Koplev, Lystrup and Vorm [3] examined the cutting process of unidirectional carbon fibre reinforced plastics in directions perpendicular and parallel to the fibre orientation. A series of quick-stop experiments was carried out to examine the area near the tool tip. The authors stated that the machining of CFRP consists in a series of fractures, each creating a chip. In the following years there were extensive contributions improving knowledge regarding composites and the most frequent associated problems.

One of the most common problems relates to the need of drilling without delamination. Several studies on this subject have been reported, and it is therefore now possible to envisage a drilling strategy that keeps delamination risk at a minimum. Davim and Reis [4], studied the effect of cutting parameters on specific cutting pressure, delamination and cutting power in carbon fibre reinforced plastics. The authors concluded that feed rate has the greater influence on thrust force, so damage increases with feed. Hocheng and Tsao [5], conducted several practical experiments to support the benefit of using special drills instead of twist drills. In this work, the authors concluded that thrust force varies with drill geometry and with feed rate which allows for the use of higher feed rates if adequate drill geometry is selected. Durão *et al.* [6], confirmed the influence of appropriate drill geometry selection on delamination reduction, as well as the advantage of the use of a pilot hole strategy.

Zitoune *et al*. [7] investigated the influence of the machining performance during drilling of sandwiched composites using various dimensions of double cone drill. In the experimental work presented, the authors concluded that it is possible to reduce the thrust force during drilling by using a double cone drill in a multi-material aeronautic component (copper mesh/CFRP laminate/carbon-epoxy fabric layer).

Another option frequently referred to in order to avoid delamination, is the use of a backup plate. The effects of using a backup plate on delamination are well known in the composites industry. This drilling strategy is always a good option when the opposite side of the plate is accessible, which sometimes is not the case, mainly in field work as those involved in maintenance or repair are well aware. The use of a backup plate allows for drilling with higher feed rates, and consequently with higher thrust forces, as critical thrust force for delamination onset is also higher [8].

Non-conventional machining processes were also addressed in several published papers, see [9,10]. In [9] the authors concluded that although the surface roughness of machined walls was higher when drilling with Abrasive Water Jet, the outcomes for mechanical resistance are dependent not only on the damage extension but also on the type and mode of loading. In [10] the authors used a Thermographic Damage Criterion (TDC) based on heat dissipation to assess the effect of the machining process on the mechanical behavior of CFRP plates, concluding that the choice of the machining process has a paramount impact on the mechanical behavior of CRFP plates.

After the drilling operation is completed, some damage in the region around the hole boundary is present; delamination being the most serious as it can reduce the load carrying capacity of the joint. Sometimes, due to the nature of the material, this damage is not detected by visual inspection, leading to the need of non-destructive testing (NDT) to assess the soundness of the parts. This effect can be diminished by a correct choice of tool geometry and/or cutting parameters. In general, it is accepted that a drilling process that reduces the thrust force exerted by the drill chisel edge can prevent the delamination risk.

In this work, some of the typical problems associated with drilling operations are studied and discussed. The main focus is the experimental determination of the critical thrust force for the delamination onset and its correlation with the damage extension caused by the drilling operation. For this purpose, delamination onset tests according to the procedure presented by Lachaud *et al.* [11] are performed. Then, similar plates are drilled and delamination around the hole quantified using image processing and analysis algorithms on enhanced radiographic images [12]. Finally, the delamination onset results are compared with existing analytical models such as the Hocheng-Dharan model [13]. The results found in this work show the importance of adequate knowledge of the material properties when establishing a suitable drilling strategy for the machining of composite materials.

This work also intends to contribute towards setting adequate and sound testing standards to assess the mechanical strength loss of assembly joints in composite parts.

## 2. Results and Discussion

The results of the maximum thrust force variation according to drill geometry and feed rate were largely published and discussed see, for example, [4,5,13–15]. According to that extensive published work, it is possible to say that, as the feed rate rises, the resultant thrust forces get higher too. It is also possible to say that different geometries cause a variation in the thrust force evolution during drilling and, consequently, on the values of the maximum force obtained during drilling, all the other factors remaining constant: plate characteristics and drill diameter. In addition, it is known that higher diameters cause larger thrust forces. However, the main focus in this work was on the damage assessment and mechanical consequences in terms of bearing resistance, so this issue is not discussed herein.

### 2.1. Double Cantilever Beam (DCB) Test Results

**Figure 1.**
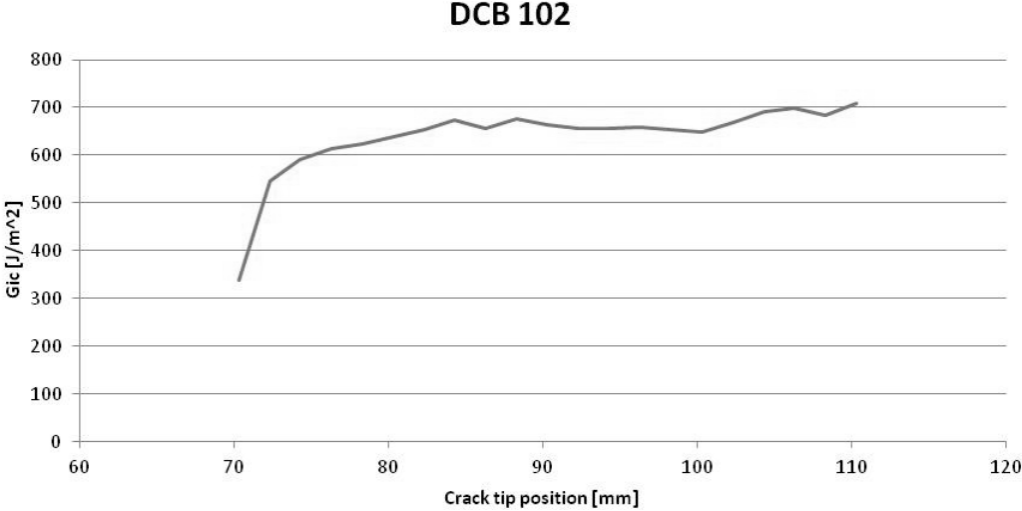
Example of a load-displacement curve from a DCB test.

The results of the DCB test are presented in Table 1, and an example of a load-displacement curve can be seen in Figure 1. The values obtained in this test are in accordance with other values found in the published literature [16,17], and were important for the subsequent mechanical testing.

### 2.2. Delamination Assessment

The delamination assessment was carried out according to the procedure described in Section 4.2. The average results concerning the unidirectional plates and all the combinations of drills and feed rates are presented in Tables 1 and 2, respectively. From these results, it is possible to conclude that, as expected, an increase in the feed rate had a direct effect in the delamination extension, for both assessment criteria. Based on these results and on the previous works referred to above, a clear connection between the thrust force results and the delamination extension can be established. The drilling conditions with less damage corresponded to the coupons associated to the lowest feed rate.

The results from the damage extension related with the tool geometry are shown in Table 2. It should be noted that, to reduce the amount of data presented, Table 1 presents average values for the three feed rates used in the experimental work.

Although the stacking sequence was not studied here, it is known that this parameter also had some effect on the delamination extension. The damage in unidirectional plates tends to be higher and extended along the direction of the fibers. This effect is less noticeable in cross-ply or quasi-isotropic plates, where the remaining drilling conditions remain unchanged. Taking into account the tool influence, the lowest values of damage extension were those obtained for the plates drilled with Brad drill. This is a special tool, designed to increase the tension of the fiber before cutting, thus enabling a clean cut and a smooth machined surface. The damage from the step drilled plates was almost equal. The carbide twist drill holes presented a damage extension that is, on the average, 10% higher. Confirming the evidence that HSS drills should not be used for drilling carbon/epoxy composites, the damage extension was always the highest, around 40% more extended damage on the average.

From these results, it is possible to state that the selection of an appropriate combination of tool geometry in relation to the characteristics of the plate is more important than the tool’s influence. This must always be kept in mind when defining the drilling conditions. Unsurprisingly, the feed rate has to be kept as low as possible, to minimize damage extension. The limit on this condition is given by the need to avoid unwanted thermal damages that result from the matrix softening and the need, in industrial terms, to ensure a reasonable number of hourly productions.

### 2.3. Mechanical Testing

**Figure 2.**
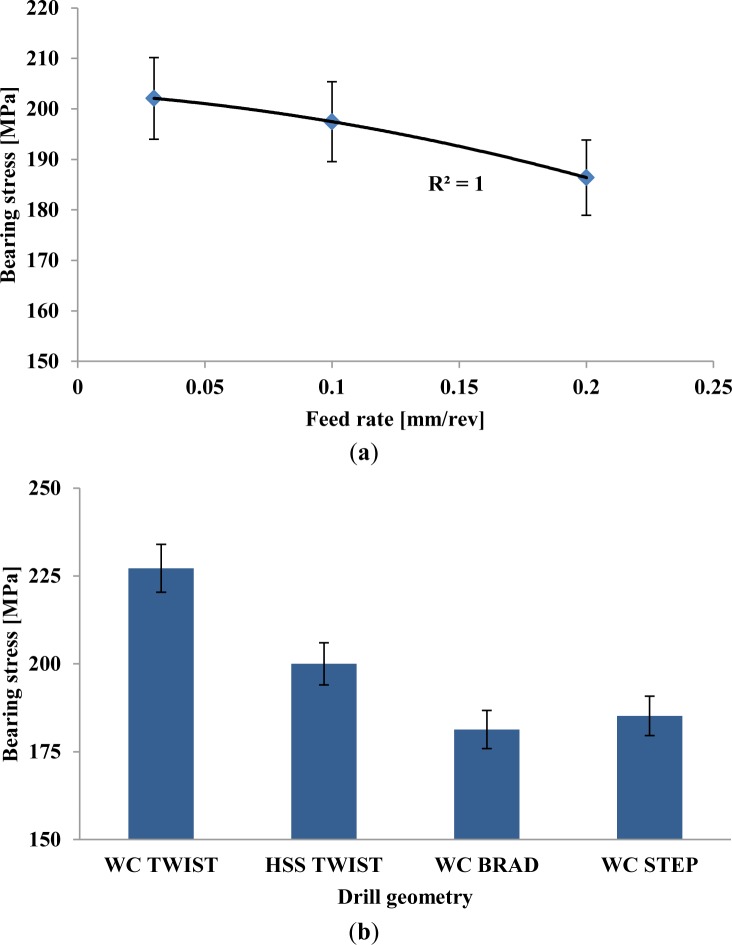
Bearing stress test: (**a**) feed rate effect on the bearing strength; (**b**) drill geometry influence on the bearing strength.

The results of the bearing stress test are shown in Figure 2, including the results for the step drill geometry.

A correlation between feed rate and mechanical loss by the bearing strength can be easily identified in Figure 2a, as one can learn from the polynomial trend line. Larger values of feed, although promoting productivity by higher output of drilled plates per hour, had an adverse effect on the damage extension, thus leading to mechanical loss. Independently of the tool used, all plates drilled with higher feed rates had lower values of mechanical resistance. From the lowest to the highest feed rate used in this experimental sequence, there was an average loss of 8% on the bearing strength result. This result means that higher feed rates should be avoided when drilling composite plates.

On the other hand, the same correlation could not be identified for the drill geometry. In spite of the larger extension of the damaged area, the plates drilled with the twist drill returned higher values of bearing strength, as observed in Figure 2b. This result suggests that the drill geometry is a key factor in damage onset and propagation. So, a good selection of the tool, combined with the feed rate can reduce the damage extension. Nevertheless, one should keep in mind that the existence of damage, such as delamination, around the hole can sometimes act as a stress relief factor increasing the force-carrying capacity of the plate. Therefore, testing results can sometimes be odd when correlating with damage extension caused by diverse drill geometries.

**Figure 3.**
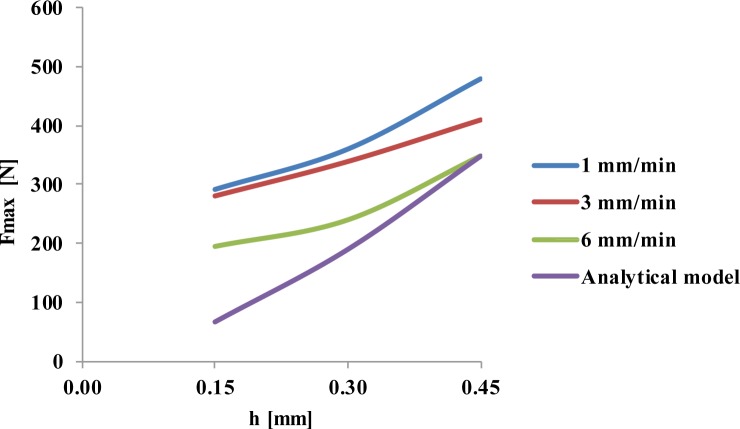
Delamination onset test results as a function of uncut thickness (*h*) and test speed—experimental values and analytical model Equation (1).

As to the delamination onset test, the results found are presented in Figure 3. These results correspond to the tests carried out using the tungsten carbide twist drill as a punch. As expected, the delamination onset load increases with the uncut thickness, according to analytical models prediction. Regarding the testing speed, there was a large scattering of the results when using the lowest speed: 1 mm/min. Finally, the experimental work was focused on the two remaining speeds: 3 and 6 mm/min, so more specimens were tested under these two speeds. It seems, from the results presented herein, that the delamination onset load tends to decrease as testing speed increases. This aspect of the testing speed will need further attention as the lower feed rates used in drilling, around 0.01 mm/rev, would correspond to a testing speed of 14 mm/min in a 1400 rpm spindle speed machine, but without the cutting action promoted by the rotating movement of the tool. There is no doubt that it is important to establish a range of acceptable testing speeds to enhance and strengthen the conclusions from this experimental technique.

A fourth line was added into the graph of Figure 3, corresponding to the theoretical values obtained when using Equation (1) and the measured properties of the plates. The experimental results are higher than the analytical model values. As the uncut thickness increases, the values tend to be equal. This shows the conservative options of the model, also pointed out by the authors in [13] enabling a cautious selection of parameters and corresponding thrust forces during drilling thus reducing the risk of delamination onset during machining.

Finally, when trying to establish a correlation between the tool geometry and the delamination onset load, no clear trend was found, and some results were surprisingly unstable, giving the general idea of the existence of outlier factors that could influence the results. The dispersion was higher than normally expected when testing composite materials and the trends were not steady when changing from one drill geometry to the other. Some of the factors that could affect this result could be: inadequate testing speed, misalignment of the punch drill axis with the hole axis, friction between the hole walls and the drill or even the evidence that this test will not give any difference regarding the tool geometry. A possible reason is that the piercing action carried out by the drill mainly depends on the plate interlaminar fracture toughness, and not on drill tip geometry and so the shape of the contact zone between drill and plate will not be of any importance.

Further testing is needed to establish some conclusions on the testing speed influence together with a review of the testing device concept and compare the results when using a smaller diameter of the punching tool—or larger diameter of the blind hole—to prevent friction.

## 3. Delamination in Composite Materials

### 3.1. Delamination Mechanism

Delamination is a damage that is likely to occur in the interlaminar region, along the contact plan between the adjacent layers in laminate parts. It therefore depends not only on fibre nature but also on resin type and respective properties such as the interlaminar fracture toughness, the elastic modulus or the Poisson ratio. The delamination mechanisms are divided into push-down and peel-up, according to on which laminate side it occurs: drill exit or entrance, respectively.

**Figure 4.**
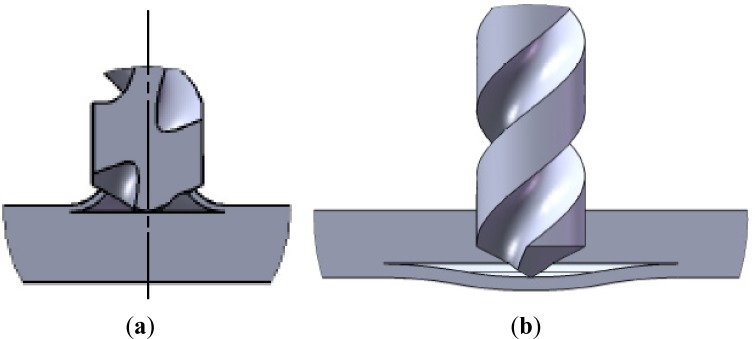
Delamination mechanisms: (**a**) peel-up delamination at entrance; (**b**) push-down delamination at exit.

Peel-up is caused by the cutting force pushing the abraded and cut materials to the flute surface. Initially, the cutting edge of the drill will abrade the laminate. As the drill moves forward, it tends to pull the abraded material along the flute, and the material spirals up before being effectively cut. This action creates a peeling force upwards that tends to separate the upper laminas of the plate (Figure 4a). This peeling force is a function of tool geometry and friction between tool and workpiece [13].

Push-down is a consequence of the compressive thrust force that the chisel edge of the drill always exerts on the workpiece. The laminate under the drill tends to be drawn away from the upper plies, breaking the interlaminar bond in the region around the hole. As the drill approaches the end of the laminate, the uncut thickness becomes smaller and the resistance to deformation decreases. At some point before the laminate is totally penetrated by the drill, the loading exceeds the interlaminar bond strength and delamination occurs (Figure 4b). A suitable tool geometry that lowers the thrust force can reduce the delamination damage [13].

A recent advance on machining strategy was given by Schulze *et al.* [18] minimizing the damage by directing the process forces towards the center of the workpiece. This is achieved through a combined process of circular and spiral milling on a three-axial machining center. According to the authors, the advantages of this process still require further research.

The works published by Hocheng and Tsao have contributed to the understanding of the delamination mechanism associated with different drilling conditions, like drill geometry [5,19], the use of a core drill [14] or the influence of using an exit back-up plate on delamination depending on drill geometry [8]. In [5,19], distinct drill bits are compared for drilling-induced delamination. The different drill geometries considered in these works are the twist drill, the saw drill, the candle stick drill, the core drill and the step drill. In [14], only the core drill was studied, showing that grit size and feed rate are the most important parameters for delamination reduction and should be kept low. According to the authors, there are advantages in using special drill bits for composites drilling. The traditional twist drill provides a reduced threshold of the thrust force for delamination onset when compared to other geometries. Concerning these geometries, the higher threshold feed rate at the delamination onset was obtained with the core drill, followed by the candle stick drill, saw drill and step [5].

A comparative study regarding distinct drill geometries and feed rate was presented in [15]. The authors assessed the thrust force, surface roughness and delamination extension for five different drill geometries and two feeds, concluding that the twist drills are well suited for carbon/epoxy plates drilling. However, only one drill diameter was considered. A study on the cutting variables on the thrust force, torque, quality of hole and chip was presented in [20]. A comparative study on the machinability of metallic materials and fiber-reinforced composites and the influence of various factors on the machinablity of these materials was presented in [21].

### 3.2. Damage Models

The analysis of delamination during drilling in composite materials using fracture mechanics has been developed and different models presented. The models referred to herein are based on the study of carbon/epoxy laminates, although other materials, like glass/epoxy or hybrid composites are also suitable for their application. The main focus on carbon/epoxy laminates can be explained by the fragile nature of the carbon fibers, when compared with glass fibers that are less troublesome in machining study. The delamination mechanisms are assumed to be modeled by linear-elastic fracture mechanics (LEFM), considering the laminate structure of composites, its high modulus of elasticity in direction 1 and the failure in delamination form.

From known models, the one that is most referred to is the Hocheng and Dharan delamination model [13]. The authors studied the onset of delamination in two different situations: push-down at exit and peel-up at entrance. The first one is the result of the compressive thrust force that the drill exerts on the uncut plies of the laminate, whose thickness is reduced as the drill advances. At some point, the loading exceeds the interlaminar bond strength of the material, and delamination occurs (Figure 4b).

According to the authors, the applicability of LEFM to composite has been previously discussed and confirmed, provided that crack growth is collinear and the crack is in a plan of material symmetry.

In this model, the critical thrust force for delamination onset, *F*_crit_, is related to properties of the unidirectional laminate, such as the elastic modulus, *E*_1_, the Poisson ratio, υ_12_, the interlaminar fracture toughness in mode I, *G*_Ic_, and the uncut plate thickness, *h*:

Fcrit=π8GIcE1h3123(1−υ122) (1)

A comprehensive summary of the steps towards free-delamination holes can be found in [22]. Starting from the model presented in Equation (1), Zitoune and Collombet [23] developed a numerical model and compared the results obtained with the two processes—numerical and analytical. The validation of the numerical model is carried out thanks to quasi-static punching tests in an experimental assembly very similar to the one presented here. A good correlation has been noticed between the numerically calculated efforts and those which were experimentally obtained.

### 3.3. Damage Extension Assessment

**Figure 5.**
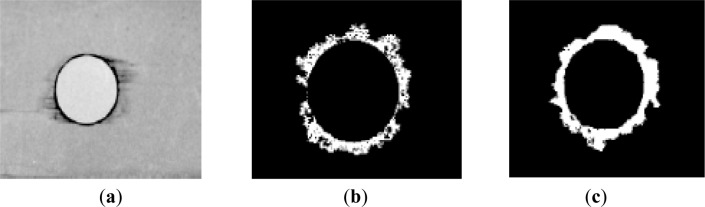
Damage evaluation: (**a**) radiography [26]; (**b**) ultrasonic C-Scan [28]; (**c**) computerized tomography [28].

After laminate holes are drilled, it is important to establish criteria to compare the delamination extension caused by different machining processes in an easy way. Damage extension can be evaluated through NDT. Some examples are: the use of a tool maker’s microscope [4], ultrasound techniques [24], acoustic emission [25], enhanced radiography [26,27] (Figure 5a), C-Scan [28] (Figure 5b) or Computerized Tomography (CT) [28–30] (Figure 5c). In all these methods, the main goal is to obtain images representing the hole surrounding areas that can be further analyzed and measured, mainly for diameters and areas.

**Figure 6.**
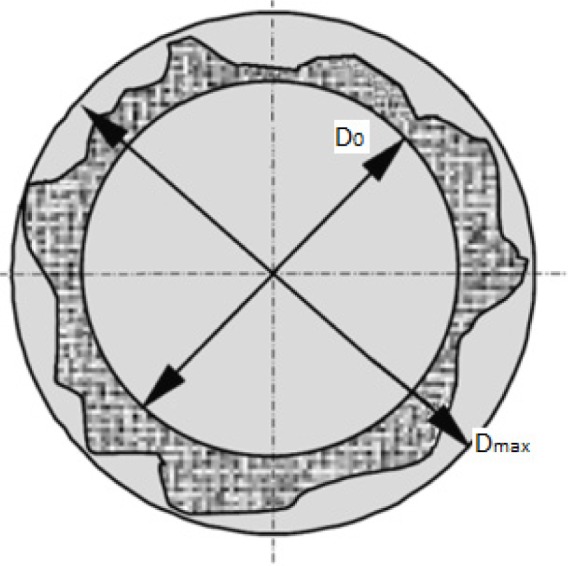
Measurement of the maximum delaminated and hole diameters.

Then, it is possible to carry out the quantification of the damaged region to calculate a factor that numerically expresses the damaged region extension and shape. Chen [31] presented a comparing factor that enables the evaluation and analysis of delamination extent in laminated composites. That ratio was called the delamination factor, *F*_d_; and it was defined as the quotient between the maximum delaminated diameter, *D*_max_; and the hole nominal diameter, *D*_0_ (Figure 6):

$$F_{d}=D_{\max}/D_{0}$$ (2)

In the experimental work presented in [31], the author examined the effects of tool geometry and cutting parameters as well as of tool wear on the delamination factor. Two types of drills were used: a carbide drill and a HSS drill. The damage zone was evaluated by using radiographic non-destructive inspection, and the results showed a near-linear relationship between the delamination factor and average thrust forces for both drill materials. The author also concluded that the thrust force increased when the drill point angle increases and that the helix angle did not have a significant effect on this force. Also, the tool flank wear causes an increase of the delamination factor, as the thrust force increases with the tool wear.

Although the feed rate has a strong influence on the thrust force; the cutting speed has not shown a significant effect on that force. Finally; the author noticed the absence of a built up edge during carbon/epoxy machining.

Mehta *et al.* [32] have suggested a different ratio with the same purpose, named damage ratio, *D*_RAT_; defined as the ratio of the hole peripheral damage area, *D*_MAR_; to the nominal drilled hole area, *A*_AVG_, *i.e.*,:

$$D_{\text{RAT}}=D_{\text{MAR}}/A_{\text{AVG}}$$ (3)

This hole damage evaluation method is based on the existence of damage images from C-Scan and pixel counting of the digitized damage area, as described in [32], or from digitized radiographs [33]. In spite of the interesting approach of this criterion, it will not be used in this work.

One limitation of Chen’s criterion is related with situations when the delamination involved is not circular, but presents breaks and cracks. In such cases, the values of the delaminated area are more appropriate for the damage quantification. Based on this, Davim *et al.* [34] presented a novel approach known as the Adjusted Delamination Factor, *F*_da_:

$$F_{\text{da}}=\alpha \frac{D_{\max}}{D_{0}}+\beta \frac{A_{\max}}{A}$$ (4)

where *A*_max_ is the area corresponding to the maximum delaminated diameter *D*_max_; and *A* is the hole nominal area. In this new criterion, the first term is the conventional delamination factor and a second term was added to take into account the damaged area contribution, and the parameters α and β are used as weights. Their sum always equal to 1 (one).

## 4. Experimental Work and Discussion

### 4.1. Composite Plates Production and Drilling

For the experimental work, a batch of 300 × 300 mm^2^ carbon/epoxy plates using prepreg CC160 ET 443, SAATI, Appiano Gentile, Italy, with 24 layers were produced. The unidirectional plates intended for DCB testing had a 70 mm Teflon^®^ insert, Wrightlon, AirTech, Huntington Beach, CA, USA, at the extremities to act as a pre-crack. The plates were then cured for one hour under 300 kPa pressure and 130 °C, followed by cooling. Final plate thickness was equal to 4 mm. Then, the plates were cut into test coupons of 135 × 36 mm^2^ for the bearing tests and the delamination onset test experiments and in coupons with dimensions according to the mechanical test specifications—DCB test, tensile test. Calcinations tests according to EN ISO 1172:1988 [35] were performed and the result showed a fiber content of 64%. Tensile tests according to ASTM D3039-08 [36] were also carried out and the plate’s properties, presented in Table 3, were found (see Section 2.1).

The experimental work initiated with the drilling of the laminate plates for thrust force monitoring, delamination measurement by enhanced radiography and automated computational algorithms of image processing and analysis and mechanical tests. Then, the composite coupons were tested according to ASTM D5961-10 [37]—Procedure A. Finally, the results of the delamination damage assessment were correlated with the bearing stress and delamination onset tests results.

The drilling operation was carried out in a 3.7 kW DENFORD Triac Centre CNC machine (Denford, West Yorkshire, UK). As it has been previously identified, feed rate is crucial compared to spindle speed in the development of thrust forces [26]. The cutting speed was kept constant and equal to 2800 rpm and the feed rate had three levels: low feed rate of 0.03 mm/rev, intermediate feed rate equal to 0.10 mm/rev and high feed rate of 0.20 mm/rev. These cutting parameters were selected according to previous published works [15,26,33] as well as the tool manufacturer’s recommendation. A tool diameter of 6 mm was used combined with the three drill geometries: twist, Brad and step. Details on the drills can be found in [15]. The tool selection is in line with past papers published by the same authors, see [6,12,15,26,33]. Twist drill is still the most common drilling tool used in almost every tool shop, Brad drill is a commercial drill normally available in tool manufacturers’ catalogue and step drill is an experimental prototype tool that has been studied and developed by the first author along the last years. Although other geometries are normally referred to in published papers [3–5,7,8,11,14,18–22,28] they are not often found in current tools products.

The experimental sequence described herein does not correspond to a complete factorial or to a Design of Experiment plan as there were some limitations on the tool availability and number of test coupons. Considering HSS tools, there are no Brad or step tools or other geometry for composites material drilling. This option was to highlight the worst possible case in terms of experimental results and to enable us to demonstrate the consequences for mechanical test results when comparing the results of HSS and WC twist drill. So, the main target is the comparison of three different tool geometries.

For the DCB test specimens according to ASTM D5528-13 [38], it was necessary to bond piano hinges at the edge of the Teflon insert. To enable the bonding operation, both the piano hinges and the extremities of the specimens were prepared with sandpaper, carefully cleaned and bonded using industrial adhesive Araldite^®^ 2012 (Everberg, Belgium). Then, one of the sides of each specimen was painted white and marked with vertical lines every 2 mm with a special mark at the end of the Teflon^®^ insert (Everberg, Belgium).

The specimens for the delamination onset test were blind drilled using an end mill and stopping the machining cycle to have different uncut thicknesses according to the height corresponding to one to four plies, that is to say, 0.15 mm, 0.30 mm, 0.45 mm and 0.60 mm.

### 4.2. Delamination Assessment

After the drilling process, the delaminated region around each drilled hole was evaluated using enhanced digital radiography. To generate a suitable image contrast, the plates were first immersed in di-iodomethane for approximately 15 to 20 min. Then the radiographic images were acquired using a 60 kV, 300 kHz Kodak 2100 X-Ray system associated with a Kodak RVG 5100 digital acquisition system (Kodak Corp., Rochester, NY, USA). The exposition time was set to 0.125 s.

**Figure 7.**
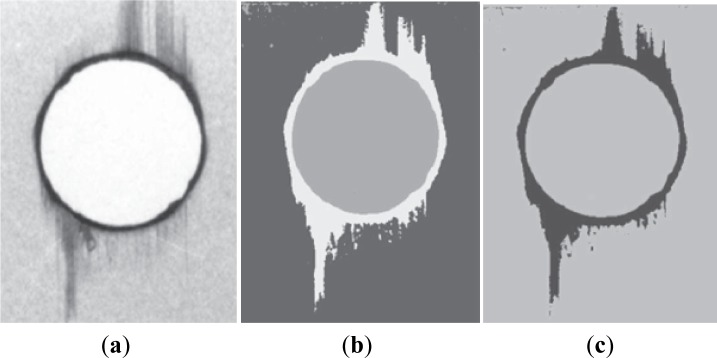
Pipeline of the computational processing of a radiographic image: (**a**) original image; (**b**) image segmented by using a neuronal network; (**c**) identification of the delamination region.

Each radiographic image was computationally processed to identify and characterize the regions of interest: hole region, delaminated and non-delaminated regions. The hole region corresponds to the central area, the delaminated region consists on a dark border around the machined hole, and the non-delaminated regions are lighter areas located outside the damaged region (Figure 7) [26].

The final goal of the image processing and analysis performed was to automatically measure the damaged diameters and areas in each radiographic image. This was achieved by using in the image segmentation step a neuronal network with 1 input layer with 3 neurons, 1 hidden layer with 7 neurons [39], 1 output layer with 3 neurons, and the logistic function [40] as the neurons activation function, which was trained using the back propagation learning algorithm [41]. The inputs of the network were each image pixel’s value, and the output was the correspondent segmentation region. Further details of the neuronal network used can be found in [42]. After the identification of the three regions presented in an input image, the related diameter and area values were computed: the diameters were calculated by searching for the longest diagonal within the delaminated region, and the areas by summing up the pixels within the associated regions.

The values obtained from the radiography images can be used to determine the delamination factor, *F*_d_, [31], the Damage Ratio, [32], and the adjusted delamination factor, *F*_da_, [34], according to Equations (2), (3) and (4) (see Section 3.3).

So, taking advantage of the computational techniques of imaging processing and analysis, the adjusted delamination factor was used in this work for damage assessment.

### 4.3. Mechanical Testing

In all the tests performed in this work, a Shimadzu AG-X/100 kN Universal Testing machine (Shimadzu, Kyoto, Japan) equipped with a Trapezium X software for data registration was used.

The DCB tests according to ASTM D5528 -13 [38] were carried out at a testing speed of 5 mm/min both for pre-crack and final crack opening. The values of time, force and displacement were registered by the Shimadzu machine system, and a synchronization method was developed to ensure data correlation between the force and crack opening. The values of the energy release rate were calculated using the Modified Beam Theory method with a 5% offset for the initiation values of the interlaminar fracture toughness in Mode I, G_Ic_.

The mechanical test carried out to assess the damage effects on plate resistance in the area of the mechanical joint was the “Bearing test” according to ASTM D5961M-10 [37]. This test was used to assess the effect of the delamination extension on the mechanical properties of the drilled plates in the joint area. Test coupons of 135 × 36 mm^2^ were cut and drilled under the same experimental conditions.

**Figure 8.**
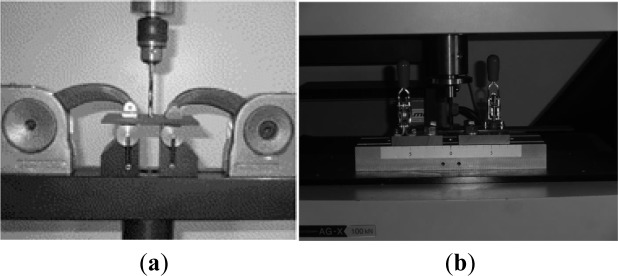
Delamination onset test: (**a**) device presented in [11]; (**b**) experimental work.

Finally, for the delamination onset test, a device based on the work of Lachaud *et al.* [11] was designed and built, see Figure 8. With the purpose of evaluating the several possible effects of test variables, three different testing speeds were used: 1 mm/min, 3 mm/min and 6 mm/min. These testing speeds were combined with the plates previously drilled with blind holes machined using twist, Brad and step drills. Details on these drills can be found in [15]. To carry out the test, a perpendicular load was applied using a drill as a punch (Figure 8). The drill movement was only linear, without rotation.

## 5. Conclusions

Carbon fiber reinforced laminates were drilled with the objective of comparing the performance of three different tool geometries regarding the bearing stress and delamination onset load. Relevant results considered for assessment were the delamination extension and the mechanical strength by the bearing stress test and delamination onset test. A batch of plates was prepared to perform DCB tests. According to the experimental findings, it was possible to make the following conclusions:

- As expected, when dealing with composite materials, some dispersion on the results was found;
- The results of the DCB test to determine the interlaminar fracture toughness were within the predicted values;
- The synchronization system developed for the DCB test allows for an easy trace of the load-displacement curve and crack opening measurement, reducing error hazard;
- The feed rate influence is well known and the results confirmed that higher feeds correspond to higher delamination extension and lower values of bearing resistance. So, in conventional drilling, the feed rates should be kept as conservative as possible;
- In the delamination onset test, as the uncut thickness increases, the delamination onset load also increases. This was an expected outcome.
- Also in the delamination onset test, as testing speed increases, the delamination onset result tends to decrease. For this test, there is a need to establish a standardized speed and to enhance the procedure for test results robustness:
- The influence of the punch geometry (drill) has to be thoroughly investigated as no clear trend was found.
- In future works, the effect of alternative stacking sequences, like cross-ply or quasi-isotropic, deserve some attention.
- Future development of the present work will have the purpose of adding analytic and FEM models for the simulation of drilling with delamination onset determination.

## Figures and Tables

**Table 1.** Results of the delamination criteria for the three feed rates studied.

Feed Rate (mm/rev)	Delamination Factor (*F*_d_)		Adjusted Delamination Factor (*F*_da_)	

	Value	Standard deviation	Value	Standard deviation
0.03	1.186	0.05	1.431	0.11
0.10	1.692	0.08	1.959	0.11
0.20	2.196	0.10	2.566	0.16

**Table 2.** Results of the delamination criteria for tool geometry and tool material.

Tool geometry	Delamination Factor (*F*_d_)		Adjusted Delamination Factor (*F*_da_)	

	Value	Standard deviation	Value	Standard deviation
WC Twist	1.655	0.07	1.858	0.08
HSS Twist	2.034	0.06	1.719	0.08
WC Brad	1.523	0.12	1.695	0.14
WC Step	1.553	0.06	1.670	0.08

**Table 3.** Material properties.

Property	Fiber content (%)	R_m_ (MPa)	E_1_ (GPa)	E_2_ (GPa)	ν	GIc (N/mm)
Value	64	1700	111	7	0.29	0.419
Standard deviation	–	20	9	1	0.05	0.017

## Nomenclature

*A*: hole nominal area
*A*_AVG_: nominal drilled hole area
*A*_max_: area corresponding to the maximum delaminated diameter
*D*_0_: hole nominal diameter
*D*_max_: maximum delaminated diameter
*D*_RAT_: damage ratio
*D*_MAR_: hole peripheral damage area
*E*_1_: elastic modulus in 1-direction
*E*_2_: elastic modulus in 2-direction
*F*_crit_: critical thrust force for delamination onset
*F*_d_: delamination factor
*F*_da_: adjusted delamination factor
*G*_Ic_: interlaminar fracture toughness in mode I
*G*_IIc_: interlaminar fracture toughness in mode II
υ_12_: Poisson ratio
